# Understanding, Trusting, and Applying Scientific Insights to Improve Your Health: A Latent Profile Analysis Approach

**DOI:** 10.3390/ijerph19169967

**Published:** 2022-08-12

**Authors:** Nejc Plohl, Bojan Musil

**Affiliations:** Department of Psychology, Faculty of Arts, University of Maribor, Koroška Cesta 160, 2000 Maribor, Slovenia

**Keywords:** scientific knowledge, trust in science, health literacy, health behavior, COVID-19, latent profile analysis

## Abstract

Various leading causes of death can be prevented or delayed through informed decision-making and lifestyle changes. Previous work has, to some extent, linked such health-promoting behavior (HPB) with variables capturing individuals’ understanding of science, trust in science, and capacity to apply evidence-based information in the health context. However, empirical research on the relationship between scientific knowledge, trust in science, health literacy, and HPB is scarce. Additionally, no study has investigated whether these characteristics interact to form homogeneous, high-risk subgroups of the population. The present online study (*N* = 705) revealed that trust in science and health literacy were positively related to a wide array of HPBs (e.g., healthy nutrition, physical activity, stress management), while scientific knowledge was only positively associated with COVID-19 vaccination intention. Furthermore, the results of latent profile analyses yielded four subgroups (i.e., low, moderate, and high levels of all three variables and a varied profile exhibiting very low trust in science, low health literacy, and moderate scientific knowledge). The identified subgroups differ significantly in HPB and variables determining profile membership (e.g., political conservatism). Hence, the present study offers some guidance on which groups may be targeted with public health campaigns and how they may be designed.

## 1. Introduction

There is a growing body of literature claiming that many of the world’s leading causes of death are preventable. A classic example illustrating this point is coronary heart disease (CHD), responsible for about 9 million deaths worldwide in 2019, substantially more than the approximately 7 million deaths two decades ago [1]. Even when such statistics are adjusted for temporal changes in the age of the population, CHD is still the number one cause of mortality, killing about 83 Americans every hour [2]. According to meta-analyses focusing on CHD prevention, a significant proportion of these deaths could have been prevented or delayed by various health-promoting behaviors (HPBs) such as physical activity [3,4,5], healthy diet [6,7], and improved stress management [8,9]. Naturally, CHD is not the only major cause of death that could be reduced through improved decision-making and lifestyle changes. Some cancers are also preventable through public health interventions [10]. Moreover, even COVID-19, which has had a profound impact on society in recent years, can now be significantly mitigated due to recent developments in understanding how the disease is spread and how the public can be inoculated against the virus [11,12]. However, it is far from given that public health interventions aimed at promoting beneficial behaviors and curbing these diseases are indeed successful in influencing lifestyle choices and informing health decisions. For example, the prevalence of obesity in the United States continues to rise sharply despite numerous attempts to address this problem [13]. Similarly, recent studies conducted on large samples show that approximately a quarter of US citizens do not plan to get vaccinated against COVID-19 despite various public health campaigns highlighting the safety and efficacy of available vaccines [14]. This number is even higher in some other countries; for example, as of July 2022, almost half of the Romanian population and two-thirds of the Bulgarian population have not yet been fully vaccinated against COVID-19 [15].

It is well-known that decisions related to health, such as the decision to be vaccinated, are notoriously complex and influenced by many factors [16], leading to several models that aim to explain the variability in individuals’ behavior. One of the most widely cited and empirically supported is the Health Belief Model, which consists of four factors that determine the likelihood of engaging in HPB—perceived susceptibility to a condition, perceived severity of contracting an illness, perceived benefits of performing actions available to reduce the threat, and perceived barriers to undertaking the recommended behavior. These four components are influenced by cues to action (e.g., mass media campaigns) as well as the modifying factors (e.g., personality and knowledge) [17]. In the present paper, we focus on three modifying factors that could facilitate or hinder engaging in HPB. Specifically, we argue that people are less likely to benefit from public health campaigns and adopt behaviors that would benefit their health if they: (1) do not understand the general science (or evidence) on which they are based; (2) do not trust the primary source of evidence (i.e., scientists); (3) have difficulties obtaining, processing, and applying health information specifically.

The first factor, scientific knowledge or scientific literacy (1), is often regarded as a synonym with public understanding of science and generally includes components such as knowledge of the substantive content of science and the ability to distinguish it from nonscience [18,19]. Although empirical studies of its relationship with HPB are scarce, often indirect, and inconclusive [20,21], improved decision-making in the context of health is hypothesized to be an important micro-level benefit of high scientific literacy, as decisions in this domain often demand some understanding of science and its processes [19,22]. The second factor, trust in science (2), refers to the extent to which individuals believe that scientific claims are honest and accurate reflections of researchers’ work [23]. While this variable has traditionally been used as an outcome, not as a predictor, recent studies related to COVID-19 identified trust in science as one of the critical variables that determine individuals’ compliance with COVID-19 prevention guidelines [24,25]. The third factor, health literacy (3), is set explicitly in the health context (not science in general) and should not be equated with scientific literacy. Instead, it generally includes aspects such as basic health knowledge, applying this knowledge to make health decisions, as well as skills and motivation to find, use, and assess the validity of health information [26]. Higher health literacy is consistently associated with better health outcomes, including higher indulgence in HPB, and is, thus, considered one of the critical public health goals [27,28,29].

Although all three factors described above could play an essential role in determining health decisions and lifestyle, the existing body of knowledge has several shortcomings. First, only a few empirical studies [20,21,24,25] have investigated to what extent scientific knowledge and trust in science are individually related to a wide array of HPBs. Second, due to the lack of studies exploring all three factors simultaneously, in a single study, it remains relatively unclear whether they exhibit similar patterns of correlations with HPB (and other variables of interest), how they compare in terms of the strength of association with HPB, as well as how they relate to each other. Third, though the variables are theoretically related yet distinct, it is not clear if (or how) they interact to form subgroups of individuals who are at exceptionally high risk in terms of their health outcomes.

In the present study, we hence aim to determine the bivariate associations of scientific knowledge, trust in science, and health literacy with HPB (*Objective 1*). Furthermore, we aim to employ a person-centered approach, specifically latent profile analysis (LPA), to determine profiles based on complex patterns of relationships between all three factors (i.e., scientific knowledge, trust in science, and self-reported health literacy) in order to identify the groups of individuals who are at risk in terms of their health-related outcomes (i.e., health-promoting lifestyle including nutrition, physical activity, stress management, health responsibility, compliance with COVID-19 prevention guidelines, and COVID-19 vaccination intention; *Objective 2*). Lastly, we compare subgroups based on different variables traditionally treated as predictors of indicators used, such as education level, religiosity, political conservatism, and conspiracy ideation, e.g., [21,30,31], and determine the key predictors of profile membership (*Objective 3*). This knowledge could help us unravel at-risk groups that may be specifically addressed with public health campaigns in the future. Furthermore, this study’s results could provide critical knowledge on how such campaigns should be designed to overcome the main barriers to adopting HPBs in these groups. In other words, the study’s findings may be used to develop targeted and tailored approaches to public health messaging that address at-risk groups who are less likely to benefit from universal approaches. Such knowledge and approaches may be vital amid the (likely lasting) COVID-19 pandemic, characterized by considerable variability in compliance with the prevention guidelines, e.g., [32], and vaccine hesitancy within certain pockets of the population, e.g., [33].

## 2. Materials and Methods

### 2.1. Participants

A total of 1066 participants from the United States started to fill out the survey. After excluding participants who dropped out (*N* = 143) and participants who were not attentive enough (i.e., failed two or all three attention checks; *N* = 218), the final sample included 705 participants. Slightly less than two-thirds of the sample were female (61.0%), followed by participants identifying as male (37.9%) or non-binary (1.1%). The age of participants ranged from 18 to 93 years, with a mean of 44.92 years (*SD* = 17.80). In terms of their educational level, most respondents had a higher secondary education (51.8%), followed by those with a bachelor’s degree (24.1%), a master’s degree (8.9%), primary education or less (7.5%), lower secondary education (6.0%), and a doctoral degree (1.7%). Additionally, the majority of participants had annual incomes of USD 25,000–49,999 (26.8%), USD 10,000–24,999 (21.6%), or USD 50,000–74,999 (15.9%).

The final sample of participants did not differ from inattentive participants in gender (χ^2^(2) = 0.82, *p* = 0.664) and education (*t* = −1.71, *p* = 0.088, *d* = 0.13), but was significantly older (*t* = −9.63, *p* < 0.001, *d =* 0.63) and had higher income (*t* = −2.03, *p* = 0.043, *d* = 0.16). Comparisons with those who dropped out are impossible as these participants did not provide us with demographic data.

### 2.2. Measures

#### 2.2.1. Scientific Knowledge

The apprehension of basic scientific facts was measured using 11 objective questions (e.g., “*Electrons are smaller than* atoms”; for all items, see Appendix A) drawn from previous studies examining general scientific knowledge that is not particularly religiously contested [34,35,36]. Participants were able to answer with “*True*”, “*I don’t know*”, or “*False”* and their responses were scored such that correct answers were assigned one point and false or “*I don’t know*“ answers were assigned zero points. The short objective test had satisfactory internal consistency (α = 0.68).

#### 2.2.2. Trust in Science

Trust in science was measured with the Trust in Science and Scientists Inventory, which captures a general level of trust in science and scientists [37]. While the scale originally consists of 21 items answered on a 5-point agreement scale ranging from one (“*Strongly disagree*”) to five (“*Strongly agree*”), we used a shorter version with 14 items that has previously exhibited an acceptable measurement model and good internal consistency [24]. This was also the case in the present study (α = 0.92). Example item: “*Scientific theories are trustworthy”*.

#### 2.2.3. Health Literacy

Individuals’ self-reported knowledge, motivation, and competencies to access, understand, appraise, and apply health information were measured using the short form of the European Health Literacy Survey Questionnaire, which consists of 16 items (HLS-EU-Q16; [38]), such as “*How easy is it for you to understand information in the media on how to get healthier*?”. These items were answered on a 5-point scale ranging from “*Very difficult*” to “*Very easy*”. The scale exhibited great internal consistency (α = 0.92).

#### 2.2.4. Conspiracy Ideation

The 15-item Generic Conspiracist Beliefs scale was used to measure individual differences in generic conspiracist ideation [39]. The scale includes subscales on government malfeasance, extraterrestrial cover-up, malevolent global conspiracies, personal wellbeing, and control of information, which are answered on a 5-point scale ranging from “*Definitely not true*” to “*Definitely true*”. As in previous studies (e.g., [39]), these subscales were combined into the general second-order factor (e.g., “*The spread of certain viruses and/or diseases is the result of the deliberate, concealed efforts of some organization*”). The scale showed great internal consistency (α = 0.95).

#### 2.2.5. Health-Promoting Lifestyle

To measure the extent to which adults engage in a health-promoting lifestyle, we used the Health-Promoting Lifestyle Profile II (HPLP II; [40]). While the scale originally contains 52 items and six subscales, we only used four of them in the present study, namely: nutrition (9 items, e.g., “*Choose a diet low in fat, saturated fat, and cholesterol*”; α = 0.82), physical activity (8 items, e.g., “*Take part in leisure-time (recreational) physical activities (such as swimming, dancing, bicycling)*”; α = 0.88), health responsibility (9 items, e.g., “*Report any unusual signs or symptoms to a physician or other health professional*”; α = 0.83), and stress management (8 items, e.g., “*Get enough sleep*”; α = 0.81). The subscales can also be combined into a total health-promoting lifestyle score (α = 0.94). Answers to all the items are given on a frequency scale from 1 (“*Never*”) to 4 (“*Routinely*”).

#### 2.2.6. COVID-19 Compliance

Compliance with COVID-19 prevention guidelines was measured using an 11-item scale, which tackles different COVID-19 preventive behaviors outlined by the World Health Organization, Centers for Disease Control and Prevention, and European Center for Disease Prevention and Control (e.g., “*Frequently washing your hands with soap and water for at least 20 s*”; [24]). Participants were asked to what extent they act per these guidelines, to which they responded using a 4-point frequency scale from “*Not at all*” to *“To a great extent*”. The scale exhibited great internal consistency (α = 0.90).

#### 2.2.7. COVID-19 Vaccination Intention

We also asked participants whether they have already received the COVID-19 vaccine (“*Yes*” or “*No*”). Those who answered with “*No*” received one additional question aimed at measuring their COVID-19 vaccination intention. The response format for this question was a 5-point agreement scale ranging from “*Definitely not*” to “*Definitely yes*”. In the analyses, we only used the intention variable, as only 133 participants (18.9%) had already received the COVID-19 vaccine at the time of the study.

#### 2.2.8. Sociodemographic Data

Various sociodemographic data were collected during the study (gender, age, education level, annual income, religiosity, and political conservatism). Education level was assessed by asking participants to mark their highest level of education, with response options ranging from “*Primary education or less*” (1) to “*Doctoral degree or equivalent*” (6). Religiosity was assessed with one item; specifically, participants were asked to express their level of agreement with the statement “*I am very religious*” on a 7-point agreement scale from “*Strongly disagree*” to “*Strongly agree*”. Finally, political conservatism was measured using the following two questions: “*How would you describe your political outlook with regard to (1) social/(2) economic issues?*”, answered on a response scale from 1 (“*Very liberal*”) to 7 (“*Very conservative*”; e.g., [41]). As the questions were highly correlated, they were treated as two indicators of political conservatism (α = 0.94).

#### 2.2.9. Attention Checks

Three attention checks were inserted into the survey, approximately every 1–2 pages. Specifically, we used the directed questions, which tell the participants to give specific answers (e.g., “*This is a control question. Mark “Agree” and move on”*; [42]). Attention checks were scored by summing the number of mistakes each subject made on these items to create scores ranging from 0 to 3.

### 2.3. Procedure

The study was conducted in February 2021. Participants were recruited randomly using Prime Panels, a compilation of opt-in online research panels with their own participant pool. The provider reports that it has an overall pool of more than 50 million survey participants who are invited to participate in studies via email and dashboards and compensated for their time. According to previous examinations, Prime Panels generally provide diverse participants (in terms of age, religiosity, education, and political attitudes) who are less used to classic protocols compared to some other panel providers [43]. Anyone aged 18 years or above was eligible to participate in the online study. While no specific sample size calculations were performed before the study, we aimed to surpass the median sample size observed in past research that employed similar analyses (*N* = 494; [44]). We also considered a general rule of thumb that a minimum sample size of about 500 participants is needed to accurately identify the latent profiles [45].

Participants were first given a brief description of the study, including basic information about the study’s objectives and methodology, and were informed that their participation was anonymous, voluntary, and could be terminated at any time. After consenting to participate in the study, participants were asked to fill out the questionnaire battery described earlier. For individuals who decided to participate in the study, the study procedure took approximately 10–15 min to complete. In return for their time, participants received USD 1.50. The study was conducted in accordance with the Declaration of Helsinki. Ethical review and approval were not required for this study according to the national and institutional guidelines.

### 2.4. Statistical Analyses

Statistical analyses were performed using IBM SPSS Statistics 26.0 and Mplus 8.0. In the first step, participants who dropped out before the end of the study and inattentive participants (i.e., those who failed more than one attention check) were excluded from all analyses. Next, we analyzed the prevalence and randomness of missing data among the remaining participants. As the results showed that missing data are rare (none of the items had a response rate of less than 98.9%) and likely missing completely at random (as shown by Little’s Missing Completely at Random test; χ^2^(5587) = 5213.63, *p* = 1.000), we replaced missing values using the expectation-maximization algorithm. We then performed normality tests, calculated skewness and kurtosis, and checked for outliers that might affect the results. As these analyses did not reveal any issues, we calculated basic descriptive statistics and used the parametric Pearson correlation coefficient to investigate associations between variables.

Following these steps, we used LPA with maximum likelihood with robust standard errors (MLR) as the estimator to identify subgroups of individuals characterized by similarities in their scientific knowledge, trust in science, and health literacy. The best-fitting profile solution was determined using multiple criteria. First, relative fit information criteria, specifically the Akaike Information Criterion (AIC), Bayesian Information Criterion (BIC), and Sample-Adjusted BIC (SABIC), were considered. In these cases, low values indicate a better fit. Second, we took into account the confidence with which individuals have been classified as belonging to one group or another. A standard parameter used for this is entropy, which has a conventional cut-off point of 0.80 or higher [46]. Third, we quantified specific comparisons between the model of interest and a model with one fewer class using the Bootstrapped Likelihood Ratio Test (BLRT). When the result is not significant for a model with k + 1 profiles, the solution is not superior to a k-profile solution. Fourth, parsimony and meaningfulness were also considered. For example, some sources report that when the profile includes <1.0% of the total sample size, the profile should be rejected [47]. Lastly, theoretical plausibility, which should be given priority when the fit values allow for this, was also considered [44,48].

To explore differences between the identified profiles, we performed a Pearson’s chi-squared test and several analysis of variance (ANOVA) tests. In cases when the data had unequal variances, we performed Welch’s ANOVA instead. Significant omnibus tests were followed up with post hoc tests, using Hochberg’s GT2 correction (due to considerably different group sizes) or Games–Howell correction (when the data exhibited unequal variances) to adjust for multiple testing. All of these analyses are accompanied with effect sizes, specifically eta squared (η^2^) and Cohen’s *d*. Given the observed differences in demographic and psychological factors between the profiles, we also used multinomial logistic regression to identify the key predictors of group membership.

## 3. Results

The results section includes descriptive statistics and data regarding the bivariate associations between scientific knowledge, trust in science and health literacy, and HPB (*Objective 1*; see Section 3.1). We then present the identified profiles and explain how they differ regarding HPB (*Objective 2*; see Section 3.2). Lastly, we present findings related to predictors of profile membership (*Objective 3*; see Section 3.3).

### 3.1. Descriptive Statistics and Correlations

Basic descriptive statistics (*M* and *SD)* and correlations between variables are presented in Table 1. The results show that scientific knowledge, trust in science, and health literacy are generally weakly positively correlated; the only exception is the correlation between scientific knowledge and health literacy, which is very close to zero. Furthermore, scientific knowledge correlates significantly positively with education and COVID-19 vaccination intention (weak correlations), while it is significantly negatively related to religiosity and conspiracy ideation (weak correlations). Correlations between general scientific knowledge and political conservatism, a health-promoting lifestyle, and COVID-19 compliance did not reach the conventional statistical significance threshold. Trust in science, on the other hand, is significantly positively correlated with education level, a health-promoting lifestyle, COVID-19 compliance (weak correlations), and COVID-19 vaccination intention (moderate correlation). At the same time, it is significantly negatively correlated with religiosity (weak correlation), political conservatism, and conspiracy ideation (moderate correlations). Lastly, self-reported health literacy is associated significantly positively with a health-promoting lifestyle, COVID-19 compliance, and COVID-19 vaccination intention (weak correlations). It is also significantly negatively associated with political conservatism and conspiracy ideation (negligible to weak correlations), while its associations with education and religiosity are not significant. Correlations between the three core constructs (scientific knowledge, trust in science, and health literacy) and the HPLP II subscales (nutrition, health responsibility, physical activity, and stress management) can be found in Appendix A (Table A1).

### 3.2. Identification of Profiles and Differences in Health-Related Outcomes

We investigated various fit statistics for solutions with two to six profiles (Table 2). While relative fit information criteria, entropy, and BLRT generally favor the five-profiles solution, this solution results in two small profiles (1.7% and 1.1% of the sample) with limited interpretability. Hence, we chose a more parsimonious and meaningful four-profiles solution. Among the solutions with significant BLRT values, this solution had the second-lowest AIC, BIC, and SABIC. Additionally, the entropy value is above the conventional cut-off and suggests that participants can be allocated to the correct latent profile with sufficient certainty.

Of particular importance, the four-profiles solution showed four quantitatively different profiles regarding their content (Table 3). Specifically, all three ANOVA tests showed significant differences between the extracted profiles. In the case of scientific knowledge, most post hoc comparisons were significant at the *p* < 0.001 threshold (*d* ranged from 0.48 to 0.91). The difference between the first and the second profile was significant at the *p* < 0.050 level (*d* = 0.61), while comparisons of the second and third profile (*d* = 0.15) and the second and fourth profile (*d* = 0.31) did not reveal significant differences. In the case of trust in science, all pairwise comparisons showed significant differences (*d* ranged from 3.33 to 12.04; *p* < 0.001), while health literacy differed significantly between the first and third profile (*d* = 0.32, *p* < 0.001), first and fourth profile (*d* = 1.01, *p* < 0.001), as well as third and fourth profile (*d* = 0.73, *p* < 0.001). The remaining pairwise comparisons did not yield significant results, although the comparison between the second and fourth profile also approached significance (*d* = 1.03, *p* = 0.071). Effect sizes for the remaining two comparisons (profile 1 vs. profile 2 and profile 2 vs. profile 3) were 0.16 and 0.48, respectively.

**Figure 1:**
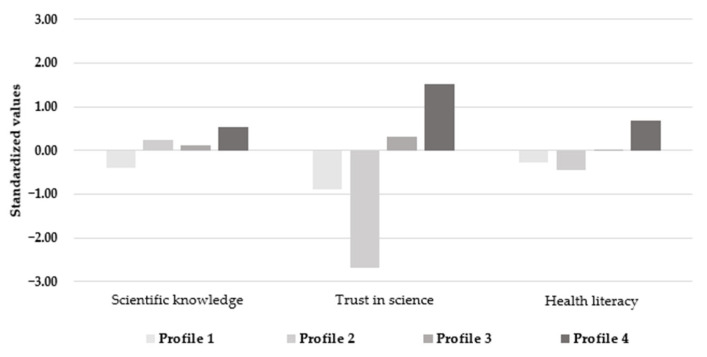
*Z*-standardized values of the indicator variables.

The first extracted group, which was medium to large in size (Profile 1; *n* = 251, 35.6%), showed low levels in all three indicators, suggesting a poor understanding of science and health information and low trust in scientists and medical experts. The second extracted group, which was small in size (Profile 2; *n* = 18, 2.6%), exhibited varied levels in the three indicators. In particular, this group exhibited a moderate comprehension of science in general but very low trust in science and low self-reported health literacy. Next, the third extracted group, which was large in size (Profile 3; *n* = 328, 46.5%), exhibited moderate levels in all three indicators. This profile can, thus, be interpreted as having a moderate understanding of science and medical information and moderate trust in scientists and medical experts. Lastly, the fourth extracted group, which was medium in size (Profile 4; *n* = 108, 15.3%), showed high levels in all three indicators, implying a high understanding of science and medical information as well as a high trust in scientists and medical experts. Figure 1 shows the standardized values of the indicator variables separately for each extracted profile, where zero refers to the mean value observed in our sample, negative values suggest that the value lies below the mean, and positive values suggest that the value lies above the mean. Larger values indicate a larger distance from the sample mean (measured in standard deviation units).

There were no differences between the extracted profiles in age (*F*(3, 701) = 1.30, *p* = 0.273, η^2^ = 0.006) nor gender (χ^2^(6) = 10.67, *p* = 0.099). More importantly, we also investigated whether the extracted profiles differ in HPB (Table 4).

Due to the violation of the homogeneity of variances assumption, we performed Welch’s ANOVA tests for all the variables presented in Table 4. As shown in the table, the four extracted profiles differed significantly in all of the studied health-related outcomes. The post hoc tests additionally revealed that the health-promoting lifestyle differs significantly between profiles 1 and 3 (*d* = 0.25*, p* = 0.013), profiles 1 and 4 (*d* = 0.61, *p* < 0.001), as well as profiles 3 and 4 (*d* = 0.39, *p* = 0.009), while the comparison between profiles 2 and 4 approached significance (*d* = 0.61, *p* = 0.077). The remaining comparisons were not significant and the effect sizes ranged from 0.08 to 0.34. Differences between profiles were even more pronounced in the case of COVID-19 compliance, where most of the pairwise comparisons were significant (*d* ranged from 0.28 to 1.70 in these comparisons). The only exception was the comparison between the first and second profile, which, however, exhibited a large effect size (*d* = 0.88, *p* = 0.104). Similarly, the profiles differed considerably in COVID-19 vaccination intention (all pairwise comparisons yielded significant results and *d* ranged from 0.39 to 2.30). Pairwise comparisons for the HPLP II subscales are presented in Appendix A.

### 3.3. Predictors of Profile Membership

In the end, we investigated whether the extracted profiles differ in variables that are traditionally understood as predictors of the indicators used in the present study (scientific knowledge, trust in science, and health literacy). The results of these analyses are presented in Table 5.

Due to the violation of the homogeneity of variances assumption, we again performed Welch’s ANOVA tests for all the variables presented in Table 5 and found that the four extracted profiles differ significantly in other variables as well. Pairwise comparisons of education level revealed significant differences between profiles 1 and 3 (*d* = 0.22, *p* = 0.040) and profiles 1 and 4 (*d* = 0.51, *p* < 0.001), while the comparison between profiles 3 and 4 approached significance (*d* = 0.25, *p* = 0.051). When comparing religiosity between profiles, only the comparisons between profiles 1 and 4 (*d* = 0.63, *p* < 0.001) and profiles 3 and 4 (*d* = 0.45, *p* = 0.001) yielded significant results. Post hoc tests for political conservatism mostly led to significant results, with *d* ranging from 0.37 to 1.11; the only exceptions are comparisons between profiles 1 and 2 (*d* = 0.24) as well as profiles 2 and 3 (*d* = 0.60). Lastly, all pairwise comparisons of conspiracy ideation were significant and *d* ranged from 0.45 to 1.63.

The variables presented in Table 5 were also used as predictors of profile membership in a multinomial logistic model. The results showed that the model containing the predictor variables outperforms the model containing only the intercept (χ^2^(12) = 196.96, Nagelkerke *R*^2^ = 0.27, *p* < 0.001). A more detailed look reveals that education (χ^2^(3) = 14.64, *p* = 0.002), religiosity (χ^2^(3) = 7.87, *p* = 0.049), political conservatism (χ^2^(3) = 38.04, *p* = < 0.001), and conspiracy ideation (χ^2^(3) = 92.40, *p* = < 0.001) are all significant contributors to the model. The key parameter estimates are reported in Table 6.

## 4. Discussion

This study aimed to (1) investigate the associations between general scientific knowledge, trust in science, health literacy, and HPB; (2) examine if scientific knowledge, trust in science, and health literacy interact to form homogeneous subgroups of the population and test whether these subgroups exhibit different levels of health-related risk; (3) determine the factors that predict profile membership.

Analyses pertaining to the first objective revealed that while the indicators used were generally weakly positively associated, we, interestingly, observed only a negligible correlation between scientific knowledge and health literacy. This finding might seem surprising at first, as health education and science education are somewhat interconnected and both include some form of knowledge. However, health literacy is more content-specific and includes many additional elements (e.g., skills to find and use health information; [26]). Moreover, scientific knowledge is conventionally measured with objective tests [49], while health literacy instruments vary in their approach and design, with many of the widely used instruments capturing self-reported, subjective health literacy [50]. These different approaches to measuring the two constructs could further contribute to a low correlation coefficient between the variables.

Furthermore, the results revealed that trust in science and health literacy are positively related to all of the included HPBs, while scientific knowledge is only significantly associated with COVID-19 vaccination intention. These results suggest that both trust in science and health literacy are related to HPB in a domain-general way, significantly extending the existing literature that linked trust in science only with COVID-19-related health outcomes [24,25] and further outlining the importance of fostering health literacy among the public [27]. A more detailed look reveals that both variables exhibit a similar strength of associations with a health-promoting lifestyle, while our data show that trust in science is a stronger correlate of COVID-related outcomes than health literacy. This is somewhat surprising as health literacy is thought to be one of the key drivers of evidence-based decision-making in the health context (even, or especially, during the time of COVID-19) [51] and further highlights the critical role of trust in science. On the other hand, scientific knowledge seems to be more domain-specific. According to our results, it is significantly associated with COVID-19 vaccination intention (even more so than health literacy) but shows only a negligible relationship with other HPBs. Further research is needed to identify health decisions in which general scientific knowledge is indeed beneficial.

Regarding the second objective, the findings we present suggest that individuals can be classified into distinct profiles based on their level of trust in science, scientific knowledge, and health literacy. Although some of the parameters involved in deciding on the number of profiles suggested a solution with five profiles, two of these profiles included approximately 1.0% of the total sample size and were not particularly theoretically meaningful. As suggested by other authors, the theoretically best-fitting solution with acceptable fit values was given priority instead [44]. Hence, the final solution consists of four subgroups, which differ in the levels of indicators used and HPB.

In particular, the largest subgroups show moderate levels of all three indicators and low levels of all three indicators, followed by a profile with high levels of all three indicators, and the smallest profile, which exhibited moderate knowledge of science but low self-reported health literacy and very low trust in science. Such results imply that for the majority of the population, all three variables that can be understood as modifying factors in the Health Belief Model [17] are similarly pronounced and appear in bundles; for example, low trust in science is generally accompanied by low scientific knowledge and low health literacy. Furthermore, among the three uniform profiles (low, moderate, and high levels of all three indicators), the profile of individuals with low scientific knowledge, trust in science, and health literacy exhibits the lowest levels of HPB. This is generally in line with the previous literature, which theoretically or empirically linked the three individual indicators with HPB and other health-related outcomes [22,24,28]. Interestingly, the additional varied profile, which is very small in size, is equally or even more (in the case of COVID-19-related variables) at-risk than the uniformly low profile even though these individuals exhibit moderate levels of scientific knowledge. Such results imply that scientific knowledge alone may not be a sufficient driver of health decisions. On the other hand, trust and health literacy may be more important as moderate and high levels of these two variables are consistently linked with favorable health-related decisions.

Lastly, analyses, comparing the profiles based on their education level, religiosity, political conservatism, and conspiracy ideation, which were performed to address the third objective, provide additional support for the validity of the identified profiles. For example, political conservatism, religiousness, and conspiracy thinking are well-known predictors of trust in science (e.g., [30,31]). In line with this, our results show that the first two profiles, characterized by low or very low trust in science, consist of individuals who are more religious, politically conservative, and prone to conspiracy ideation compared to the remaining profiles. On the other hand, education level has previously been associated with scientific knowledge [21]. As a result, education level was highest in profiles 2 and 4, which exhibit the highest level of scientific knowledge. These findings are further supported by the regression analyses, which show that all four variables are significant predictors of profile membership and that their predictive value differs between specific comparisons in line with previous research.

### Limitations

As the data were collected online, via an online panel, some of the disadvantages of online panels, such as panel bias (i.e., panelists are sometimes very experienced in filling out surveys), apply to our study. However, it should be noted that Prime Panels participants are generally less exposed to classic protocols compared to participants on other similar platforms [43]. Moreover, the study sample is not representative of the general population in terms of demographic variables; while the sample was heterogeneous, it slightly diverges from the overall US population in some aspects. For example, women were somewhat overrepresented in the sample (61.0% versus 50.7% in the general population), and so were individuals aged 25–54 (55.5% versus 49.7% in the general adult population), 65 or above (20.3% versus 17.5% in the general adult population; [52]), and individuals with a college degree (24.1% versus 16.8% in the general population; [53]). Additionally, future studies could benefit from a larger sample size; as the smallest profile only includes 2.6% of the sample, it contains—based on the current sample size—less than 25 cases, which is not ideal for LPA [47]. Sample sizes of approximately 1000 participants would, thus, be needed to fulfill this criterion and make it easier to generalize the findings.

In addition to limitations related to sampling, the study’s disadvantage is its cross-sectional nature, making it impossible to form causal inferences. Such a design may also contribute to common method bias; as all data were collected at the same time and using the same method (i.e., self-report), the relationships between variables may, to some degree, be artificially inflated. However, based on the Harman’s single factor score, which loads all items used in the study onto a single factor, this is unlikely for our study; total variance explained by a single factor is 12.8%, which is below the recommended threshold of 50.0% [54]. Future research may still investigate this further and test the observed associations in a longitudinal setting or using objective data (e.g., physical activity data collected by smartphone apps). Lastly, it is worth noting that the present study did not account for all social cognition variables linked to the measured outcomes, such as social norms. Therefore, more studies with additional variables that might explain some of the relationships and differences observed in this study are needed.

## 5. Conclusions

Although our study had limitations, the results contribute significantly to the literature on the role of scientific knowledge, trust in science, and health literacy in explaining various HPBs, including those related to the recent COVID-19 pandemic. In particular, the results reveal that trust in science and health literacy are both associated with a health-promoting lifestyle and compliance with COVID-19 guidelines, while scientific knowledge is not significantly associated with these two health-related outcomes. On the other hand, all three proposed factors are associated with COVID-19 vaccination intention, with trust in science being a particularly strong correlate. Moreover, we were able to distinguish between four subgroups of individuals based on their scientific knowledge, trust in science, and health literacy. We found three profiles with uniformly low, moderate, and high levels of these variables and a varied profile that exhibits a moderate level of scientific knowledge but low to very low levels of the remaining two variables. The results consistently show that the varied profile and the uniformly low profile exhibit lower levels of various HPBs compared to the remaining profiles. Additionally, both high-risk profiles include participants who are more politically conservative and high in conspiracy ideation compared to the lowest-risk profile (i.e., Profile 4).

While these results are interesting in a descriptive, theoretical sense, they also offer some practical implications for broader societal-level efforts and for designing public health campaigns. Specifically, they suggest that deliberate efforts toward fostering trust in science (e.g., facilitating a better understanding of the motives and methods of science among the general public) and health literacy (e.g., empowering individuals with critical health literacy skills) are needed. Furthermore, our results point to specific subgroups that should be targeted with efforts encouraging HPB and offer some guidance on how such efforts could be designed. In particular, public health messaging may benefit from being directed toward and tailored to individuals who are low in trust in science and/or health literacy. This includes providing reliable, timely, relevant, simple, easy-to-access, and easy-to-use information, as well as enrichening public health messages with information regarding scientists’ expertise, limitations, and their independence from politics [51,55,56].

## Figures and Tables

**Table 1:** Basic descriptive statistics and correlations between variables.

	*M * (*SD*)	1.	2.	3.	4.	5.	6.	7.	8.	9.
1. Scientific knowledge	0.55 (0.23)	-								
2. Trust in science	3.56 (0.73)	0.25 ***	-							
3. Health literacy	3.14 (0.50)	0.04	0.27 ***	-						
4. Education	3.26 (1.02)	0.22 ***	0.12 **	0.07	-					
5. Religiosity	3.91 (2.18)	−0.10 *	−0.21 ***	0.00	0.06	-				
6. Political conservatism	3.97 (1.76)	−0.05	−0.36 ***	−0.08 *	−0.02	0.35 ***	-			
7. Conspiracy ideation	2.86 (0.96)	−0.20 ***	−0.43 ***	−0.11 **	−0.10 **	0.08 *	0.16 ***	-		
8. Health prom. lifestyle	2.21 (0.55)	0.06	0.20 ***	0.25 ***	0.27 ***	0.17 ***	−0.05	−0.02	-	
9. COVID-19 compliance	3.43 (0.59)	0.01	0.28 ***	0.25 ***	0.01	−0.06	−0.22 ***	−0.17 ***	0.21 ***	-
10. COVID-19 vac. intention ^a^	3.35 (1.60)	0.20 ***	0.41 ***	0.11 **	0.21 ***	−0.08	−0.32 ***	−0.36 ***	0.12 **	0.37 ***

Notes. ^a^ *N* = 572 as some participants had already received the COVID-19 vaccine. * *p* < 0.050, ** *p* < 0.010, *** *p* < 0.001.

**Table 2:** Fit indices for profile solutions.

Number of Profiles	LL	FP	AIC	BIC	SABIC	Entropy	BLRT (*p*)	Smallest Profile Size
2	−1210.31	10	2440.62	2486.21	2454.45	0.447	<0.001 ***	42.4%
3	−1181.76	14	2391.51	2455.33	2410.87	0.713	<0.001 ***	1.1%
4	−1151.75	18	2347.50	2429.55	2372.40	0.825	<0.001 ***	2.6%
5	−1137.26	22	2318.51	2418.79	2348.94	0.856	<0.001 ***	1.1%
6	−1134.23	26	2320.46	2438.97	2356.42	0.870	1.000	0.1%

Notes. *** *p* < 0.001.

**Table 3:** General scientific knowledge, trust in science, and health literacy: profile means.

	Profile 1 (*n* = 251)	Profile 2 (*n* = 18)	Profile 3 (*n* = 328)	Profile 4 (*n* = 108)		
	*M* (*SD*)	*M* (*SD*)	*M* (*SD*)	*M* (*SD*)	*F*	η^2^
Scientific knowledge ^a^	0.46 (0.23)	0.60 (0.20)	0.57 (0.20)	0.67 (0.23)	27.91 ***	0.107
Trust in science ^b^	2.92 (0.27)	1.60 (0.38)	3.80 (0.26)	4.68 (0.23)	1571.40 ***	0.873
Health literacy ^c^	3.00 (0.48)	2.92 (0.88)	3.15 (0.45)	3.48 (0.47)	26.64 ***	0.104

Notes. ^a^ Classic ANOVA *F*(3, 701) was used, ^b^ Welch’s ANOVA *F*(3, 75.60) was used as the data exhibited unequal variances, ^c^ Welch’s ANOVA *F*(3, 74.41) was used as the data exhibited unequal variances. *** *p* < 0.001.

**Table 4:** Differences between profiles: HPB.

	Profile 1 (*n* = 251)	Profile 2 (*n* = 18)	Profile 3 (*n* = 328)	Profile 4 (*n* = 108)		
	*M* (*SD*)	*M* (*SD*)	*M* (*SD*)	*M* (*SD*)	*F*	η^2^
Health-promoting lifestyle ^a^	2.10 (0.53)	2.06 (0.58)	2.23 (0.50)	2.44 (0.63)	9.06 ***	0.046
Nutrition ^b^	2.05 (0.60)	2.01 (0.73)	2.20 (0.57)	2.39 (0.68)	7.57 ***	0.036
Health responsibility ^c^	2.04 (0.61)	1.93 (0.64)	2.18 (0.56)	2.41 (0.69)	8.67 ***	0.043
Physical activity ^d^	1.98 (0.72)	1.94 (0.85)	2.10 (0.73)	2.26 (0.90)	3.10 *	0.015
Stress management ^e^	2.32 (0.62)	2.38 (0.59)	2.43 (0.57)	2.72 (0.71)	8.44 ***	0.044
COVID-19 compliance ^f^	3.32 (0.64)	2.73 (1.01)	3.48 (0.52)	3.66 (0.43)	14.48 ***	0.075
COVID-19 vaccination intention ^g^	2.73 (1.52)	1.13 (0.52)	3.65 (1.46)	4.22 (1.43)	104.42 ***	0.164

Notes. ^a^ *F*(3, 75.54), ^b^ *F*(3, 75.25), ^c^ *F*(3, 75.76), ^d^ *F* (3, 75.41), ^e^ *F*(3, 76.31), ^f^ *F*(3, 75.28), ^g^ *F*(3, 82.35). * *p* < 0.050, *** *p* < 0.001.

**Table 5:** Differences between profiles: other variables.

	Profile 1 (*n* = 251)	Profile 2 (*n* = 18)	Profile 3 (*n* = 328)	Profile 4 (*n* = 108)		
	*M* (*SD*)	*M* (*SD*)	*M* (*SD*)	*M* (*SD*)	*F*	η^2^
Education level ^a^	3.07 (0.98)	3.50 (0.79)	3.30 (1.08)	3.56 (0.89)	7.97 ***	0.028
Religiosity ^b^	4.30 (2.11)	4.39 (2.77)	3.91 (2.10)	2.95 (2.18)	9.78 ***	0.042
Political conservatism ^c^	4.50 (1.63)	4.89 (1.96)	3.89 (1.65)	2.86 (1.80)	23.44 ***	0.100
Conspiracy ideation ^d^	3.18 (0.71)	3.80 (0.75)	2.80 (0.93)	2.12 (1.07)	40.06 ***	0.158

Notes. ^a^ *F*(3, 79.51), ^b^ *F*(3, 75.36), ^c^ *F*(3, 76.64), ^d^ *F*(3, 76.96). *** *p* < 0.001.

**Table 6:** Summary of parameter estimates for variables predicting profile membership.

	*B*	*SE B*	*Wald*
*Profile 1*			
Education	−0.47 **	0.14	11.58
Religiosity	0.18 **	0.07	7.71
Political conservatism	0.49 ***	0.09	31.99
Conspiracy ideation	1.18 ***	0.15	62.54
*Profile 2*			
Education	−0.11	0.26	0.17
Religiosity	0.16	0.13	1.50
Political conservatism	0.61 ***	0.16	14.69
Conspiracy ideation	2.21 ***	0.37	36.04
*Profile 3*			
Education	−0.24	0.13	3.64
Religiosity	0.13 *	0.06	4.48
Political conservatism	0.30 ***	0.08	14.40
Conspiracy ideation	0.69 ***	0.13	28.25

Notes. Profile 4 (high levels of all three indicators) is the reference category. * *p* < 0.050, ** *p* < 0.010, *** *p* < 0.001.

## Data Availability

Aggregated data are available upon reasonable request.

## Appendix A

### Appendix A.1. Items Used to Measure Scientific Knowledge

1. The center of the Earth is very hot.
2. All radioactivity is manmade.
3. It’s the father’s gene that decides whether the baby is a boy or a girl.
4. Lasers work by focusing sound waves.
5. Electrons are smaller than atoms.
6. Antibiotics kill viruses as well as bacteria.
7. The Sun goes around the Earth.
8. All human-made chemicals can cause cancer.
9. Astrology has some scientific truth.
10. The oxygen we breathe comes from plants.
11. Radioactive milk can be made safe by boiling it.

### Appendix A.2. Correlations between Trust in Science, Scientific Knowledge, Health Literacy, and HPLP II Subscales

**Table A1:** Correlations with HPLP II subscales.

	*M * (*SD*)	1.	2.	3.	4.	5.	6.
1. Scientific knowledge	0.55 (0.23)	-					
2. Trust in science	3.56 (0.73)	0.25 ***	-				
3. Health literacy	3.14 (0.50)	0.04	0.27 ***	-			
4. Nutrition	2.17 (0.61)	0.09 *	0.18 ***	0.18 ***	-		
5. Health responsibility	2.16 (0.61)	−0.01	0.20 ***	0.27 ***	0.62 ***	-	
6. Physical activity	2.08 (0.76)	0.06	0.11 **	0.14 ***	0.62 ***	0.57 ***	-
7. Stress management	2.44 (0.62)	0.06	0.18 ***	0.24 ***	0.62 ***	0.59 ***	0.60 ***

Notes. * *p* < 0.050, ** *p* < 0.010, *** *p* < 0.001.

### Appendix A.3. Pairwise Comparisons between Profiles: HPLP II Subscales

Results of pairwise comparisons, performed with the Games–Howell post hoc test, revealed significant differences in *nutrition* between profiles 1 and 4 (*d* = 0.54, *p* < 0.001) and profiles 1 and 3 (*d* = 0.26, *p* = 0.012), while the difference between profiles 3 and 4 approached significance (*d* = 0.32, *p* = 0.055). The remaining pairwise comparisons were not significant, with *d* ranging from 0.07 to 0.55.

Similarly, we observed significant differences in *health responsibility* between profiles 1 and 4 (*d* = 0.58, *p* < 0.001), profiles 1 and 3 (*d* = 0.24, *p* = 0.019), profiles 2 and 4 (*d* = 0.70, *p* = 0.037), and profiles 3 and 4 (*d* = 0.39, *p* = 0.014). The remaining pairwise comparisons were not significant and *d* ranged from 0.18 to 0.44.

*Physical activity* differed significantly only between profiles 1 and 4 (*d* = 0.36, *p* = 0.028). The remaining pairwise comparisons were not significant, with *d* ranging from 0.06 to 0.36.

Lastly, pairwise comparisons revealed that *stress management* differed significantly between profiles 1 and 4 (*d* = 0.62, *p* < 0.001) and profiles 3 and 4 (*d* = 0.48, *p* = 0.001), while the remaining comparisons did not yield significant results. Effect sizes for the remaining comparisons ranged from 0.09 to 0.49.
